# Metagenomic Identification and Characterization of Novel Vitamin B_12_ Synthesizers from the Rumen of Beef Cattle Fed High-Lipid Inclusion Diets

**DOI:** 10.3390/microorganisms13112617

**Published:** 2025-11-18

**Authors:** Angel Martinez, Benoit St-Pierre

**Affiliations:** Animal Science Complex, Department of Animal Science, South Dakota State University, Box 2170, Brookings, SD 57007, USA; angel.rodriguez@sdstate.edu

**Keywords:** rumen, cattle, lipid, triglycerides, microbiome, microbiota, bacteria, 16S rRNA, metagenomics, vitamin B_12_, ethanolamine

## Abstract

Beef production in intensive systems requires optimal nutrition to maximize growth and profitability. While triglycerides contain twice the energy per unit weight compared to polysaccharides, they are not nearly as commonly used as a supplemental source of energy compared to starch, largely in part due to their negative effects on rumen physiology when their inclusion levels are too high. To gain further insights into the response of rumen microbial communities to elevated dietary lipid levels, we took advantage of rumen samples collected as part of a previously published study that tested high inclusion (4% and 8%) of tallow or linseed oil in beef cattle as part of a 5 × 5 Latin square design, with corn used as a base dietary ingredient. Using a 16S rRNA gene-based profiling approach, two uncharacterized candidate rumen bacterial Operational Taxonomic Units (OTUs), referred to as Bt-995 and Bt-1367, were found to be in higher abundance in rumen samples collected from steers when they were fed diets with higher inclusion of linseed oil. Using a metagenomics approach to assemble contigs corresponding to genomic regions of these OTUs, various predicted metabolic functions were found to be shared. Consistent with the dietary treatments of the original animal study, functions associated with starch utilization and triglyceride metabolism were identified. Unexpectedly, however, contig sets from both OTUs also encoded genes predicted to be involved in vitamin B_12_ biosynthesis, as well as ethanolamine utilization, a function that is dependent on vitamin B_12_ as a co-factor. Together, these results indicate that vitamin B_12_-related functions may provide an advantage to rumen bacteria under conditions of high dietary triglyceride inclusion.

## 1. Introduction

Ruminant production represents an important contributor to the livestock sector. Indeed, ruminants, such as cattle, sheep, and goats, are raised throughout the world for the production of meat and milk, as well as for a variety of other by-products in demand by consumers. In the USA, the beef industry represents a particularly critical portion of the livestock sector; for instance, beef production and processing in 2016 provided over 721,500 industry jobs and generated $167 billion in gross sales [1].

In contrast to monogastric species such as swine and poultry, ruminants have evolved to digest and assimilate plant biomass. Their ability to break down and metabolize plant fiber components such as cellulose and hemicellulose is through the activities of symbiotic microbial communities, also referred to as microbiomes, that reside in the rumen compartment of their four-chambered stomach [2]. Rumen microbiomes are made up of diverse and complex communities of bacterial, archaeal, protozoal, and fungal species which work together to metabolize feed into compounds such as short chain fatty acids (SCFAs) and amino acids that can be absorbed and utilized by the host animal to support its metabolism [3]. Additionally, ruminants rely on microbial synthesis of various co-factors that they need, such as Vitamin B_12_ [4]; this microbial product is required for the function of two critical host enzymes: Methylmalonyl coenzyme A mutase (MCM) and Methionine synthase (MS) [5]. MCM is a mitochondrial enzyme required for the entry of propionate into the tri-carboxylic cycle, an essential step for its use as a substrate for gluconeogenesis. MS is a cytosolic enzyme required for generating methionine from homocysteine, a process that also requires folate [5].

In intensive beef production systems, diets are tailored to provide optimal nutrient availability to steers during their peak growth period. During this phase, feeding ingredients that contain nutrients that are more readily digestible, such as starch, or more energy dense, such as triglycerides, can be used more effectively to maximize growth performance [6]. However, as ruminants have evolved to digest and metabolize cellulose and other components of plant fibers as their primary sources of energy, feeding high levels of starch or triglycerides can have adverse effects on rumen health and function, which can ultimately impair performance. In the case of starch, a number of strategies have been developed through decades of research to control the onset of acidosis or mitigate its negative impacts [7]. In contrast, optimizing inclusion of triglycerides has not received the same level of attention; indeed, while their energy content is nearly double per mass unit compared to polysaccharides, triglycerides have been found to be detrimental to rumen microbiota when fed in excess [8,9,10]. Research on the effects of combining both lipids and starch-rich ingredients, such as higher inclusion of saturated or unsaturated triglycerides in corn-based cattle diets, has also been limited [11].

In addition to increasing the energy density of diets [12], lipids also provide other benefits. For instance, they represent important nutrients for ruminants, playing a crucial role in reproductive and hormonal health by improving luteal function and follicular dynamics [13,14]. As they help in improving the flavor of meat products, lipids thereby also contribute to more favorable consumer acceptance and increased market value, which are critical to the profitability of the beef industry [15]. In the context of these benefits, polyunsaturated fatty acids (PUFAs) are of particular great importance and interest in cattle, as they have been found to modulate hormone levels, enhance reproductive function, as well as improve meat flavor [13,16]. Moreover, supplementing ruminant diets with PUFAs has also been linked to environmental and productivity benefits; for instance, the addition of extruded linseed oil to the diets of Holstein cows was reported to reduce methane emissions by as much as 34%, while also increasing milk production efficiency [17].

In the rumen, metabolizing dietary lipids starts with lipases, as these enzymes release glycerol and free fatty acids from triglycerides through hydrolysis of ester bonds [18]. Various rumen bacterial species have been reported to possess lipolytic activity. For example, *Anaerovibrio lipolytica*, as well as other related species, have been found to produce several different types of lipases and esterases, enabling them to hydrolyze triglycerides and other esters [19]. Once released, glycerol can be readily absorbed and metabolized by rumen microorganisms, primarily by entering the glycolytic pathway to be fermented into SCFAs, which are the major source of energy for the host animal [20,21]. In contrast, there are more possible outcomes for fatty acids released by lipolysis, which is in part due to the diversity in chemical structures among these compounds, as their carbon chains can vary in length, and they can be in either a saturated (SFAs) or unsaturated (UFAs) form. These structural variations determine the potential uses of fatty acids by microbial cells, as well as their nutritional roles in animals [10]. For instance, SFAs, such as palmitic (C16:0) and stearic (C18:0) acids, are typically either stored as energy reserves or integrated into bacterial cell membrane, while their direct contribution to the animal’s energy pool through SCFA production is considered minimal [8,22,23]. In addition to being acquired from the diet, SFAs can also be synthesized de novo by rumen microorganisms. While UFAs can also be absorbed by rumen microorganisms, their assimilation can disrupt the fluidity of microbial membranes, leading to increased non-specific permeabilization [24]. To counteract this effect, rumen microorganisms typically transform UFAs into SFAs through biohydrogenation, a microbial detoxification process that neutralizes the bacteriostatic effects of UFAs, thereby reducing the risks of cell lysis [25]. Depending on the initial concentration and composition of polyunsaturated fatty acids (PUFAs) in the diet, biohydrogenation can involve various combinations of enzymatic reactions. In general, the process starts with partial hydrogenation or isomerization of linolenic acid (C18:3) and linoleic acid (C18:2) into conjugated linoleic acid (CLA), conjugated linolenic acid (CLnA) and vaccenic acid (VA), respectively; eventually, these can be further saturated to form stearic acid (C18:0). Bacterial species such as *Butyrivibrio fibrisolvens*, as well as members of the genus *Pseudobutyrivibrio*, are considered the primary agents of triglyceride hydrolysis and UFA biohydrogenation in the rumen. While protozoa do not contribute significantly to the biochemical transformation of UFAs, they effectively act as carriers of CLA and VA for the benefit of the host, first by ingesting bacteria and assimilating their content while in the rumen, then by being digested themselves as they transit to downstream gut compartments [26].

Thus, from an economic perspective, lipid inclusion in a ruminant diet can be cost-effective when used strategically [12]. However, since triglycerides can have detrimental effects on rumen health and function when their levels are too high, their inclusion in ruminant diets needs to be limited [27]. Considering our current knowledge gap on how the rumen microbiome adapts to triglyceride supplementation outside of biohydrogenation, we took advantage of samples collected during a previously conducted animal trial to investigate the composition and metabolic potential of rumen microbial communities in response to elevated dietary inclusion of saturated (tallow) or unsaturated (linseed oil) lipids [28]. The aim of this study was to identify rumen bacterial species enriched under conditions of high dietary triglycerides, and to investigate how they could thrive under these conditions. Using a Next Generation Sequencing approach, we report the identification of two candidate uncultured rumen bacterial species that were found in greater abundance when higher levels of lipids were fed. In addition to enzymatic capabilities for metabolizing triglycerides, other shared functionalities were revealed by gene annotation of metagenome assemblies for both species, such as the capacity to metabolize ethanolamine, as well as complete or near complete pathways for synthesis of vitamin B_12_. Implications of these functions in the context of triglyceride metabolism are further discussed.

## 2. Materials and Methods

### 2.1. Rumen Samples

The rumen samples used for the microbiome analysis described in this report had been collected as part of a previously published study that investigated the effects of high lipid inclusion on the performance of beef steers [28]; no separate or additional animal experimentations were performed for the microbiome analyses described in this current report. The original animal study had been approved by the South Dakota State University Institutional Animal Care and Use Committee prior to performing the trial; for more information on the original animal study, please consult the report by Blom and Brake (2018) [28]. Briefly, five ruminal cannulated steers were used as part of a 5 × 5 Latin square that was balanced for carryover effects, with treatment or control diets provided across 12-day periods (Supplementary Materials S1a). Cattle were housed in individual pens at the same facility and under standard management care, with ad libitum access to water.

Samples were collected on day 12 of each period, at a frequency of every 2 h over a 12 h period (700 to 1900 h) [28]. Rumen content was collected directly through the cannula porthole by hand, then strained with four layers of cheesecloth [28]. Equal volumes of strained fluid recovered from each timepoint were composited, then stored at −20 °C until processing for DNA extraction. All diets were based on dry-rolled corn, alfalfa hay, linseed meal and liquid cane molasses; treatment diets differed in the amount and type of lipid that was supplemented, with the addition of either 4% or 8% of either tallow (mostly saturated lipids) or linseed oil (mostly unsaturated lipids) ([28]; Supplementary Materials S1b); for more specific information on diets used in the original animal study, please consult the report by Blom and Brake (2018) [28].

### 2.2. Microbial Genomic DNA Purification and PCR Amplification of the 16S rRNA Gene

Microbial genomic DNA from individual rumen samples was extracted by a bead-beating method followed by column-based purification using the QIAamp DNA Mini Kit (Qiagen, Hilden, Germany), as previously described by Yu and Morrison [29]. The V1–V3 regions from the bacterial 16S rRNA gene were amplified via PCR using the universal primers 27F-5′AGAGTTTGATCMTGCTCAG [30] and 519R-5′GWATTACCGCGCGCGCTG [31]. PCR products were separated by agarose-gel electrophoresis, then amplified bands of expected molecular size were excised for DNA extraction using the QiaexII Gel Extraction Kit (Qiagen, Hilden, Germany). Next Generation Sequencing of gel-purified PCR products was performed by Molecular Research DNA (MRDNA, Shallowater, TX, USA) using an Illumina MiSeq 2 × 300 platform to produce overlapping paired-end reads (San Diego, CA, USA). Out of the 25 samples that were processed, four did not generate sequence data, and were not included in the analysis; these consisted of two samples from the control diet treatment (S19 and S23), one sample from the 8% tallow-supplemented diet treatment (S21), and one sample from the 8% linseed oil-supplemented diet treatment (S25).

### 2.3. Bacterial Composition Analysis

Raw DNA sequence reads were quality filtered by selecting for (i) the presence of intact 27F and 519R primer nucleotide sequences, (ii) a length of 400–580 bp, and (iii) a 1% maximum frequency of nucleotides with a Phred quality score lower than 15. Quality-filtered sequences were then aligned and clustered into operational taxonomic units (OTUs), using 4% sequence dissimilarity as a genetic distance cutoff; this threshold was selected instead of the more commonly used value of 3%, because the V1–V3 region is more variable than regions such as V3–V4, V4, or V4–V5 that are typically targeted for 16S rRNA gene-based composition analyses (for more detailed procedures and justifications, please consult the approach described by Opdahl et al. [32]). Next, 16S rRNA gene sequence artifacts were identified using the ‘chimera.uchime’ and ‘chimera.slayer’ commands from the MOTHUR open source software package (v.1.44.3) [33], as well as by using an in-house database alignment search-based approach [32]. After removal of sequence artifacts, the publicly available tools Ribosomal Database Project (RDP) Classifier (v2.14) [34] and BLAST (v2.17.0) [35] were used for taxonomic assignment of valid OTUs.

### 2.4. Metagenomics Analysis

Three samples were selected for metagenomics analysis as a strategy to investigate the metabolic potential of OTUs Bt-995 and Bt-1367. These OTUs were very abundant in rumen samples from steers fed diets with higher inclusion of unsaturated triglycerides (linseed oil), and they likely corresponded to unknown bacterial species based on 16S rRNA-based taxonomy. Two rumen samples from steers fed the 8% linseed oil-supplemented diet were selected for Bt-995, because of their high abundance (>75%) for this OTU based on 16S rRNA composition analysis. For Bt-1367, the sample with the highest representation (54.6%) was selected for analysis. As a comparison, a metagenomic analysis of a control sample with high abundance of Bt-1391, an OTU most closely related to *Sharpea azabuensis*, was also performed.

Shotgun sequence data was then generated using DNA preparations from these selected samples with an Illumina Miseq (2 × 250) platform (Molecular Research DNA, Shallowater, TX, USA). Genomic contigs were assembled separately from each short sequence read dataset using an in-house pipeline of custom-written Perl scripts [36,37]. Each contig set was annotated separately with the publicly available tools ‘Rapid Annotations using Subsystem Technology’ (RAST) [38], and ‘automated carbohydrate-active enzyme and substrate annotation’ (dbCan3). For dbCan3, annotated hits were considered valid only if they were identified as positives by all three tools (‘HMMER: dbCAN’, ‘DIAMOND: CAZy’ and ‘HMMER: dbCAN-sub’) [39]. Predicted enzyme functions from annotated genes were then assigned to metabolic pathways using an in-house custom reference based on the Kyoto Encyclopedia of Genes and Genomes (KEGG) database [40] (Supplementary Materials S2). The number of genes encoding for proteins of the large (‘LSU’) and small (‘SSU’) ribosomal subunits was used to estimate genome coverage for our assemblies.

### 2.5. Data Visualization and Statistical Analysis

A heatmap was generated in R (v4.1.1) using the heatmap.2 function from the gplots R package (v3.2.0) within RStudio (v2024.12.1.563) to visualize the relative abundance of operational taxonomic units (OTUs) across samples. Data was not scaled prior to visualization.

To assess the statistical robustness of sample clustering, bootstrap resampling was performed using the pvclust R package (v2.2). Hierarchical clustering was repeated 1000 times (*n* = 1000 bootstrap iterations) using Euclidean distance and average linkage. Clusters with AU *p*-values = 95% were considered statistically significant, and were highlighted in the dendrogram using pvrect.

## 3. Results

### 3.1. 16S rRNA Gene-Based Rumen Bacterial Composition Analysis from Steers Fed Diets with High Inclusion of Triglycerides

To gain further insight on the response of rumen bacterial communities to high inclusion of dietary triglycerides, an OTU-based composition analysis using the 16S rRNA gene was performed on samples collected from steers fed a corn-based diet supplemented with either tallow (high in saturated fatty acids) or linseed oil (high in unsaturated fatty acids) at two different levels (4% or 8%) [28]. From this analysis, a total of 3617 OTUs were identified across all samples. The twenty most abundant OTUs, whose combined representation per sample ranged between 21.3% and 97.0%, were further analyzed (Supplementary Material S3). Six of these OTUs showed at least 97% nucleotide sequence identity to their closest valid relative, indicating that they could potentially have been strains of bacterial species that have already been characterized. In contrast, the sequence identity of the other abundant OTUs to their respective closest matches ranged between 80.3% and 94.8%, suggesting that they likely belonged to candidate bacterial species that have yet to be isolated. Notably, eight of the most abundant OTUs were found to represent at least 50% of all sequence reads of a sample in at least one sample (Supplementary Material S3).

**Figure 1:**
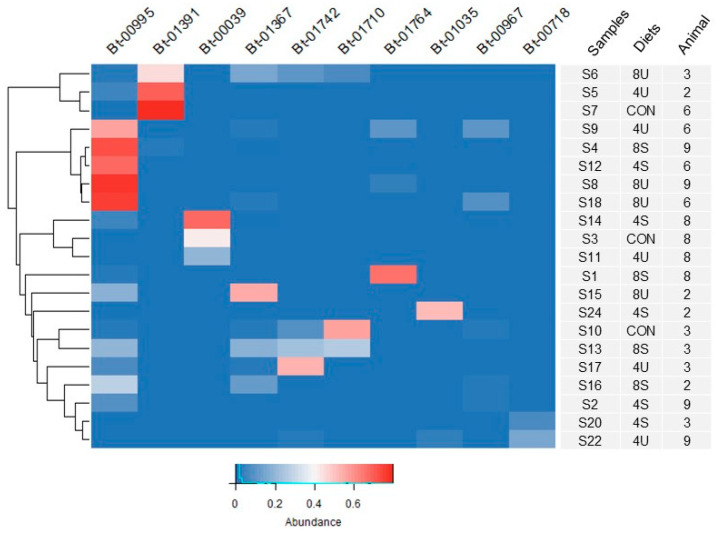
Heatmap showing the relative abundance of the 10 most abundant OTUs across samples. The corresponding animal number and dietary treatment from the original Latin square design are also indicated for each sample. Diets included: 4U (4% linseed oil, unsaturated triglycerides), 4S (4% tallow, saturated triglycerides), 8U (8% linseed oil, unsaturated triglycerides), 8S (8% saturated triglycerides), and CON (control).

As a strategy to identify rumen bacterial species that could be involved in the response to high lipid diets or favored under these conditions, we performed a hierarchical cluster analysis based on the twenty most abundant OTUs described above (Figure 1). From this analysis, OTU Bt-995 was deemed of particular interest, since it was found to be more abundant in eight samples from diets with higher inclusion of triglycerides from four of the five steers, while it was in contrast detected at much lower levels in samples from control diets (Figure 1; Supplementary Figure S1; Supplementary Material S4). Although its higher representation was limited to two of the five steers, Bt-1367 was similarly in higher abundance in samples collected from feeding diets supplemented with triglycerides compared to feeding the control diet.

### 3.2. Characterizing the Metabolic Potential of Predominant OTUs Through Assembly of Metagenomes

As OTUs Bt-995 and Bt-1367 were associated with elevated dietary triglyceride inclusion levels, we next aimed to uncover potential mechanisms that could be responsible for their apparent competitive advantage. Considering the limited sequence identity of OTUs Bt-995 (*Massiliimalia timonensis*, 86.26%) and Bt-1367 (*Anaerotruncus rubiinfantis*, 86.45%) to their respective closest valid relatives, we were unable to reliably infer their metabolic functions through comparisons with currently known species. Thus, their metabolic potential was further explored using a metagenomics approach, with the goal of assembling genomic contigs for these candidate rumen bacterial species. In order to maximize genome coverage, a strategy was devised to optimize contig length by generating metagenomic data from select samples with very high abundance of an OTU of interest; it was anticipated that a high proportion of sequence reads generated from these samples would be most likely to have originated from the genome of interest.

#### 3.2.1. Genomic Potential of Bt-1367

In the contig set for Bt-1367, 387 genes encoding proteins whose functions could be annotated were identified, as well as 1197 hypothetical genes whose functions are currently unknown. Genome coverage was estimated to be approximately 81% based on ribosomal protein representation, with the identification of 16 out of 21 proteins for the small subunit and 28 out of 33 proteins for the large ribosomal subunit. Functions associated with glucose metabolism were found to be prominent in Bt-1367. For instance, gene annotations from assembled contigs revealed the presence of 9 out of 10 enzymes necessary for glycolysis, indicating the capacity to metabolize glucose. The identification of an encoded alpha amylase provided evidence that starch may act as a source of glucose for this OTU, which was consistent with the inclusion of dry-rolled corn in the base diet in the original animal study. Bt-1367 was predicted to generate the short chain fatty acids acetate and butyrate as microbial end products that could be metabolized by the host. In addition, coding sequences for enzymes predicted to be involved in glycogen synthesis and gluconeogenesis were found, indicating a capacity to store glucose, as well as the ability to synthesize this monosaccharide from non-carbohydrate precursors. Additionally, Bt-1367 was predicted to metabolize glucose through the pentose phosphate pathway, as its assigned contig set encoded 7 out of the 8 core enzymes. One predicted function of the pentose phosphate pathway for Bt-1367 would be to serve as a source of D-ribose-5P for the production of PRPP, a precursor for the synthesis of purine nucleotides, as a result of the enzymatic activity of ribose phosphate pyrophosphokinase (EC 2.7.6.1) (Table 1; Supplementary Table S1; Supplementary Materials S5). With the identification of coding sequences for 7 out of 9 required enzymes for histidine biosynthesis, another predicted use for PRPP would be as a precursor for the de novo synthesis of histidine. Consistent with the context of this study, genes encoding key enzymes that were expected to be involved in lipid hydrolysis were identified, including monoglyceride lipase (EC 3.1.1.23) and lysophospholipase (EC 3.1.1.5). These enzymes would be predicted to cleave or release fatty acids and glycerol from triglycerides. While a gene encoding for glycerol kinase (EC 2.7.1.30) was not found in the contig set, the presence of coding sequences for glycerol-3P dehydrogenase (EC 1.1.5.3) indicated the potential for glycerol to be further metabolized through glycolysis or used as a precursor for gluconeogenesis. Notably, genes encoding enzymes necessary for metabolizing ethanolamine, one of the major structural components of phospholipids, were identified. Indeed, ethanolamine could be converted into ammonia and acetaldehyde by the enzymes EutA and ethanolamine ammonia-lyase (EC 4.3.1.7, light and heavy chains); in turn, acetaldehyde could be metabolized into ethanol by alcohol dehydrogenase (EC 1.1.1.1). In terms of possible outcomes for fatty acids released from triglycerides or phospholipids, we were able to identify a gene encoding for a putative 2,4-dienoyl-CoA reductase (EC 1.3.1.34), which could be involved in biohydrogenation of unsaturated fatty acids. As we were unable to identify genes coding for enoyl-CoA isomerase and other enzymes required for beta oxidation of fatty acids from the contig set, the ability of Bt-1367 to metabolize fatty acids may be limited to biohydrogenation based on our results [41,42].

With the identification of 8 out of 9 enzymes for synthesis of Vitamin B_2_ (Riboflavin), as well as 20 out of 28 enzymes needed for the production of Vitamin B_12_ (Cobalamin), another feature of interest for Bt-1367 was the potential to produce two essential vitamins de novo (Table 1). As riboflavin is one of the precursors for vitamin B_12_ synthesis, the Bt-1367 contig set thereby indicated a level of auto-sufficiency for this function. Identified genes included coding sequences for sirohydrochlorin cobaltochelotase (EC 4.99.1.3), an enzyme responsible for both transporting sirohydrochlorin as well as catalyzing its association with cobalt [47]. Intriguingly, the Bt-1367 contig set also included the putative ATP-biding cassette (ABC) transporters BtuF, BtuC, and BtuD, which are required for cobalamin uptake (Table 1; Supplementary Materials S6), suggesting that it may also have the ability to acquire vitamin B_12_ from the extracellular environment. As vitamin B_12_ is a required co-factor for the activity of ethanolamine ammonia-lyase, and thus for ethanolamine utilization, this pathway is consistent with the potential of Bt-1367 to utilize or metabolize phospholipids.

#### 3.2.2. Genomic Potential of Bt-995

The contig set for Bt-995 included 406 genes that could be annotated and 472 coding sequences for which a function could not be assigned (also referred to as ‘hypothetical’ proteins). Coding sequences for ribosomal proteins were used to assess coverage, which was estimated to be approximately 74%, based on identification of 26 out of the 33 expected proteins for the large ribosomal subunit, and 14 of the 21 expected proteins for the small subunit.

The metabolic capabilities of Bt-995 were assessed through functional annotation of its assigned contig set. The ability to utilize glucose was supported by the identification of seven out of ten core enzymes required for glycolysis (Table 1). The presence of coding sequences for a maltodextrin ABC transporter suggested that starch could potentially be used as a source of glucose by Bt-995 (Supplementary Materials S5). Glucose catabolism through glycolysis would result in the production of pyruvate, which could then be metabolized into the end-products lactate and acetate, based on the metabolic capabilities predicted by the Bt-995 contig set. Alternatively, glucose could also be stored as glycogen, since coding sequences for glycogen synthase (EC 2.4.1.21), as well as branching enzymes (EC 2.4.1.18; EC 2.4.1.25), were identified.

The contig set for Bt-995 suggested that this OTU would be adapted to an environment with triglycerides; indeed, coding sequences for a lipase, as well as for a putative choloylglycine hydrolase (EC 3.5.1.24) predicted to be involved in bile modification, were found. Intriguingly, the Bt-995 contig set also included functions similar to those identified for Bt-1367. For instance, copies of the eutA, eutB, and eutC genes, which would allow the utilization of ethanolamine, were identified. Enzymes predicted to be involved in vitamin B_12_ synthesis were also found in the Bt-995 contig set; however, as only a segment of the B_12_ biosynthesis pathway was identified, this OTU may need to acquire certain intermediates of the pathway from the extracellular environment. Intriguingly, the Bt-995 contig set also included the putative ATP-biding cassette (ABC) transporters BtuF, BtuC, and BtuD, which are predicted to be involved in cobalamin uptake (Table 1; Supplementary Materials S6), suggesting that it may also have the ability to acquire vitamin B_12_ from the extracellular environment.

#### 3.2.3. Genomic Potential of Bt-1391

To further our efforts in gaining a better understanding of microbial metabolic functions that may be beneficial for tolerance to high triglyceride content in ruminant diets, a metagenomics analysis was also performed for Bt-1391, an OTU that was found in higher abundance in samples from the control, non-supplemented dietary treatment. An analysis of the predicted metabolic capabilities for Bt-1391 thus aimed to offer a comparison with the functions of Bt-1367 and Bt-995. Its contig set consisted of 420 putative genes encoding proteins whose functions could be annotated, as well as 1183 coding sequences for proteins of unknown function. Based on the number of ribosomal proteins found, this contig set was predicted to represent approximately 85% of the Bt-1391 genome (30 of 33 expected proteins for the large ribosomal subunit, and 16 of 21 expected proteins from the small ribosomal subunit).

The ability of Bt-1391 to metabolize glucose was supported by the identification of coding sequences for alpha amylase, components of a predicted maltodextrin transporter, as well as for five of the ten glycolytic enzymes. End products from glucose metabolism were predicted to include formate, acetate, as well as lactate. The contig set for this OTU also indicated the potential to metabolize triglycerides, with genes encoding a monoglyceride lipase (EC 3.1.1.23) and a lysophospholipase (EC 3.1.1.5), respectively. The identification of coding sequences for glycerol-3P dehydrogenase (EC 1.1.5.3) and 2,4-dienoyl-CoA reductase NADPH (EC 1.3.1.34) indicated the potential for metabolizing glycerol and biohydrogenation of fatty acids, respectively. However, genes encoding enzymes for vitamin B_12_ biosynthesis or ethanolamine utilization were not found in the Bt-1381 dataset; this metabolic potential is consistent with the metabolic potential of *S. azabuensis* genomic assemblies currently available in public database.

## 4. Discussion

For millennia, humans have benefited from the ability of ruminants to convert plant biomass into meat, milk, and other products. To this day, ruminant livestock remain a critical component of the food chain as a sustainable source of dietary protein, because these animals feed on ligno-cellulosic plant tissues that are inedible to humans. Similarly, domesticated hindgut fermenters such as horses and donkeys that also feed on plant biomass continue to play important roles in this current era. As feed continues to be the biggest expense in ruminant production, more emphasis is being put on improving efficiency and performance. To this end, various practices have been developed over the years, including feeding ingredients that are more easily digestible, such as corn and grains, and these have allowed to improve production and profitability. Since their energy content is greater than that of polysaccharides, triglycerides would seemingly represent an attractive option to increase the energy content of ruminant diets. However, inclusion of oil or fat above critical thresholds can be detrimental to rumen physiology, because triglycerides can impair the function and survival of rumen microorganisms [10]. As the mechanisms responsible for these adverse effects remain poorly explored, we aimed to gain more insight by taking advantage of rumen samples collected as part of a previously conducted animal trial that tested the effects of higher triglyceride inclusion levels on ruminant performance [28].

In this context, this report describes two putative unknown or uncultured rumen bacterial species that were found to be more highly represented in steers that had been fed higher levels of saturated or unsaturated triglycerides compared to when they had been fed a non-supplemented corn-based control diet. Since these uncharacterized bacterial species were phylogenetically too distant from their respective closest valid relatives to reliably infer function, a metagenomics approach was used to predict their metabolic capabilities. As a comparison, the genome of an OTU found in higher abundance when steers were fed the control diet was also analyzed.

### 4.1. Hydrolysis and Biohydrogenation Potential of Bt-1367, Bt-0995 and Bt-1391

Among other metabolic capabilities, OTU Bt-1367 and Bt-1391 shared functions related to lipid metabolism, including genes encoding for lysophospholipase enzymes, which would be predicted to cleave triglycerides into glycerol and fatty acids [48]. While glycerol could be used as a substrate by pathways such as glycolysis that are commonly found in rumen microorganisms, fatty acids are typically incorporated into bacterial membranes rather than being metabolized [49]. If the fatty acids released from hydrolysis of triglycerides are unsaturated, they may subsequently undergo biohydrogenation, a protection mechanism broadly used by rumen microorganisms to counteract the toxic effects of these compounds [25]. Notably, microbial species in other environments also use this mechanism to mitigate the effects of UFAs. For instance, *Fusarium graminearum*, a pathogenic fungus that infects wheat crops, employs biohydrogenation to counteract the toxic effects of PUFAs produced by the host plant [50].

For many years, *Butyrivibrio fibrisolvens* had been reported as the main ruminal species capable of performing biohydrogenation. Since then, other rumen bacteria with this ability have been identified, including *Anaerovibrio lipolytica* and certain members of the genus *Propionibacterium* [10]. Evidence to date suggests that distinct bacterial species may be responsible for different steps during biohydrogenation [51]; thus, whether complete or only partial biohydrogenation is achieved may depend on other factors, such as location within the rumen or interspecies interactions. In support of this model, ruminal bacteria from the liquid phase have previously been reported to be associated with higher levels of C18:1-trans10 and C18:1trans11 (UFAs), which would be indicative of partial biohydrogenation, while bacteria from the solid phase were found to produce more C18:0 fatty acids (SFAs), which would be indicative of complete biohydrogenation [52].

During biohydrogenation, PUFAs are first isomerized into intermediate compounds such as CLA or CLnA, which can then be converted into stearic acid (C18:0) through the action of reductases [51,53]. Isomerases responsible for transforming linoleic acid and linolenic acid into other compounds have been well studied, largely due to high interest in CLAs and CLnAs by the food industry; indeed, these compounds have gained attention for their reported health benefits in humans, including regulation of lipid metabolism and anticarcinogenic effects [54]. As a result of our annotation analyses, 2,4-Dienoyl-CoA reductase NADPH (1.3.1.34), an enzyme reported to be involved in biohydrogenation of unsaturated fatty acids [55], was identified for two out of three OTUs of interest. However, since coding sequences for this enzyme were present in datasets from both lipid supplemented and non-supplemented diets, this metabolic activity did not appear to be selected in response to elevated inclusion of dietary triglycerides. Intriguingly, while the Bt-995 contig set did not include potential biohydrogenation enzymes, it had coding sequences for an enzyme predicted to be involved in bile transformation. Considering that biohydrogenation is a metabolic activity that requires energy [25], this result may be indicative of an alternative strategy to tolerate dietary triglycerides that is expressed by certain specialized bacterial gut symbionts. Future analyses of complete genomes for Bt-995 and Bt-1367, as well as research on isolates from these OTUs, will be necessary to confirm the extent of this potential type of metabolic specialization, as well as its impact on competitiveness in response to triglycerides.

### 4.2. Vitamin B_12_-Dependent Pathways Were Shared by OTUs That Were Predominant Under Conditions of Elevated Unsaturated Fatty Acid Supplementation

**Figure 2:**
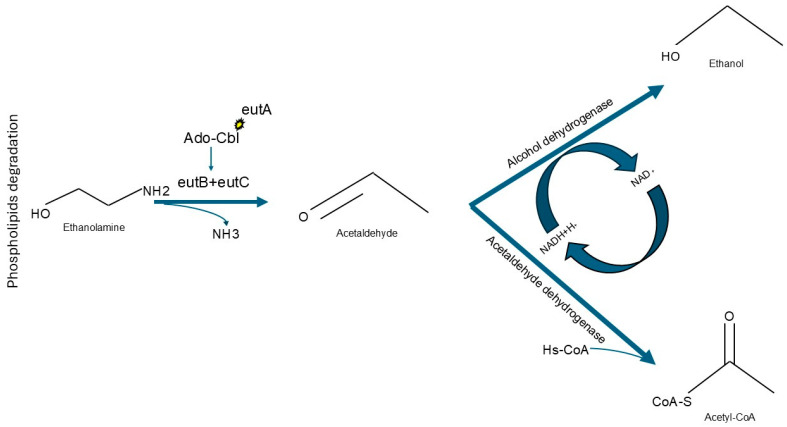
Biochemical functions of the Ethanolamine Utilization Complex. Ethanolamine can be metabolized by Ethanolamine ammonia-lyase heavy chain (eutB) and Ethanolamine ammonia-lyase light chain (eutC) when Adenosyl-Cobalamin (Ado-Cbl) is available. Ado-Cbl molecules can be regenerated by the Ethanolamine utilization protein (eutA). This reaction would generate acetaldehyde, which could then be further metabolized into Acetyl-CoA or Ethanol by Acetaldehyde dehydrogenase or Alcohol dehydrogenase, respectively.

Intriguingly, despite the extent of the phylogenetic distance between them, OTUs that were predominant in steers fed high inclusion levels of unsaturated triglycerides shared the ability to utilize ethanolamine, a function that requires vitamin B_12_ as a co-factor. In contrast, contigs for Bt-1391 did not include coding sequences for vitamin B_12_-dependent functions (Supplementary Materials S7). Ethanolamine is an organic compound that can be used by bacterial specialists as a source of carbon or nitrogen [56,57]. For example, *Salmonella enterica* can use ethanolamine as an alternative substrate when 1,2-propanediol is not available; this order of preference may be due to differences in energy yield or potential toxicity of metabolic intermediates [58,59]. While ethanolamine utilization has been primarily investigated in bacterial pathogens, other non-pathogenic bacterial species have also been reported to have this metabolic capability [60]. The ability to utilize ethanolamine can be predicted from bacterial genomes by the presence of the ethanolamine utilization (*eut*) complex, which was identified in both OTU Bt-995 and OTU Bt-1367. This cluster of genes includes *eutB* and *eutC*, which encode the subunits of ethanolamine ammonia lyase; in the presence of Ado-Cbl, a common natural variant of vitamin B_12_ [61], this enzyme is able to catalyze the conversion of ethanolamine into acetaldehyde and ammonia. Another gene in this cluster, *eutA*, encodes for an enzyme that regenerates the Ado-Cbl cofactor during ethanolamine conversion (Figure 2) [57]. Subsequently, through the action of enzymes encoded in other chromosomal loci, acetaldehyde can be transformed into either acetyl-CoA or ethanol (Figure 2; Supplementary Figure S2) [58,62].

A potential abundant source of ethanolamine for OTUs Bt-995 and Bt-1367 could have been the linseed oil used for supplementing diets during the animal trial. Indeed, oils from crops such as linseed and soybean contain high levels of phosphatidylethanolamine, a type of phospholipid that includes ethanolamine as part of its structure [63,64]. Another potential source of phosphatidylethanolamine could have been host or bacterial cell debris, as this phospholipid is also a component of mammalian and bacterial cell membranes [65].

### 4.3. Vitamin B_12_ Synthesis and Scavenging Capabilities of Bt-995 and Bt-1367

**Figure 3:**
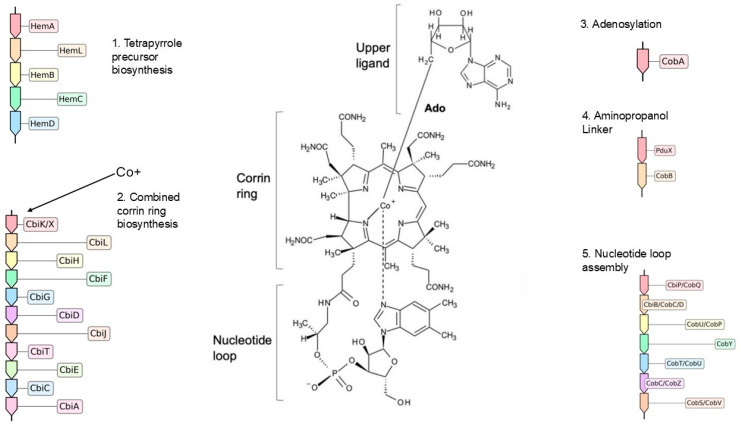
Anaerobic de novo biosynthesis of vitamin B_12_ (adenosylcobalamin, Ado-Cbl). The pathway is divided into five major modules: (1) tetrapyrrole precursor biosynthesis, (2) corrin ring biosynthesis, (3) adenosylation, (4) aminopropanol linker synthesis, and (5) nucleotide loop assembly. Enzymes are indicated by their abbreviations and EC numbers, as described in Shelton (2019) [70]. Enzyme key: HemA, glutamyl-tRNA reductase (EC 1.2.1.70); HemL, glutamate-1-semialdehyde aminotransferase (EC 5.4.3.8); HemB, porphobilinogen synthase (EC 4.2.1.24); HemC, hydroxymethylbilane synthase (EC 2.5.1.61); HemD, uroporphyrinogen-III synthase (EC 4.2.1.75); CysG, syroheme synthase/precorrin-2 dehydrogenase (EC 1.3.1.76); CbiK/CbiX, sirohydrochlorin cobaltochelatase (EC 4.99.1.3); CbiL, cobalt-factor II C20-methyltransferase (EC 2.1.1.151); CbiH, cobalt-factor III methyltransferase (EC 2.1.1.272); CbiF, cobalt-precorrin-4 methyltransferase (EC 2.1.1.271); CbiG, cobalt-precorrin 5A hydrolase (EC 3.7.1.12); CbiD, cobalt-precorrin-5B (C1)-methyltransferase (EC 2.1.1.195); CbiJ, cobalt-precorrin-6A reductase (EC 1.3.1.106); CbiT, cobalt-precorrin-6B (C15)-methyltransferase (EC 2.1.1.196); CbiE, cobalt-precorrin-7 (C5)-methyltransferase (EC 2.1.1.289); CbiC, cobalt-precorrin-8 methylmutase (EC 5.4.99.60); CbiA, cobyrinate a,c-diamide synthase (EC 6.3.5.11); CobA, ATP:cob(I)alamin adenosyltransferase (EC 2.5.1.17); PduX, L-threonine kinase, (EC 2.7.1.177); CobB, threonine-phosphate decarboxylase, (EC 4.1.1.81); CbiP/CobQ, cobyric acid synthase (EC 6.3.5.10); CbiB/CobC/CobD, adenosylcobinamide-phosphate synthase (EC 6.3.1.10); CobU/CobP, adenosylcobinamide kinase (EC 2.7.1.156); CobY, adenosylcobinamide-phosphate guanylyltransferase (EC 2.7.7.62); CobT/CobU, nicotinate-nucleotide−dimethylbenzimidazole phosphoribosyltransferase (EC 2.4.2.21); CobC/CobZ, α-ribazole-5′-phosphate phosphatase (EC 3.1.3.73); CobS/CobV, adenosylcobinamide-GDP ribazoletransferase (EC 2.7.8.26).

Since contigs from both Bt-995 and Bt-1367 were found to encode enzymes that require vitamin B_12_ [66], it indicated a need to synthesize or acquire this co-factor. The ability to synthesize or acquire vitamin B_12_ reflects an ecological adaptation to environments with limited availability of molecular oxygen, as this has been postulated to be an evolutionary conserved strategy to optimize anaerobic fermentation of small molecules [67]. Vitamin B_12_ and its variants are complex molecules consisting of a central cobalt atom that is surrounded by a cyclic tetrapyrrole structure, which is itself linked to a nucleotide loop. This co-factor is exclusively made by prokaryotes, with over 28 enzymes necessary for the complete anaerobic vitamin B_12_ biosynthesis pathway (Figure 3). In addition to gut environments, Vitamin B_12_-producing microorganisms have been described in other anaerobic habitats, including marine environments and soils [66,68,69], where they can impact species composition and metabolic outputs of microbial communities. To assess the potential of OTUs Bt-995 and Bt-1367 for de novo biosynthesis of this co-cofactor, we applied the framework established by Shelton et al. [70], which integrates publicly available genomic data alongside experimental validation. Their classification is based on the level of completeness of five key stages of the vitamin B_12_ biosynthesis pathway: (1) tetrapyrrole precursor biosynthesis (5 enzymes), (2) corrin ring biosynthesis (10 enzymes for the anaerobic pathway), (3) aminopropanol linker formation (2 enzymes), (4) adenosylation (1 enzyme), and (5) nucleotide loop assembly (7 enzymes) (Figure 3). Based on these stages, organisms can be categorized as: very likely cobalamin producers, likely producers, possible producers, tetrapyrrole precursor salvagers, cobinamide salvagers (Cbi, is an intermediate of the vitamin B_12_ biosynthesis pathway), likely non-producers, or very likely non-producers.

When this classification framework was applied to evaluate the biosynthetic capabilities of OTUs from this study, Bt-1367 was identified as a likely de novo vitamin B_12_ producer. Its contig set encoded a total of 20 enzymes spanning the full pathway: four enzymes for tetrapyrrole precursor biosynthesis, eight for corrin ring formation, one for adenosylation, one for aminopropanol and seven for nucleotide loop assembly. In contrast, the Bt-0995 contig set contained genes for most of the downstream steps, including enzymes for corrin ring formation, one enzyme for aminopropanol linker formation, as well as seven enzymes for nucleotide loop assembly; however, the Bt-995 contig set lacked genes for the early-stage tetrapyrrole precursor biosynthesis, as well as coding sequences for the adenosylation enzyme (Table 2 and Table 3; Supplementary Tables S2 and S3). Therefore, based on its predicted enzyme set, Bt-995 was classified as a cobinamide salvager, i.e., capable of synthesizing Ado-Cbl from salvaged cobinamides, which are intermediates of the Vitamin B_12_ biosynthetic pathway that can be acquired from the extracellular environment for use as precursors (Supplementary Materials S6).

Based on pathway predictions from its contig set, Bt-1367 appeared to be independent for other metabolic functions as well. Notably, it had the potential to synthesize vitamin B_2_, which could serve as a precursor for the synthesis of 5,6-dimethylbenzimidazole, the final component of functional adenosyl-cobalamin (Ado-Cbl); vitamin B_2_ also plays a key role in two-electron dehydrogenations and single-electron transfer reactions [71]. Our results suggest that elevated dietary intake of triglycerides may promote an association between full vitamin B_12_ synthesizers and partial synthesizers in the rumen of beef cattle. More research is needed to better understand the dynamics of these consortia, as well as the mechanisms of cobalamin salvaging. Notably, it has been suggested that vitamin B_12_ transporters may be acquired through horizontal gene transfer [72]. Such mechanisms could enable cobinamide-salvaging gut bacteria to use vitamin B_12_ as a cofactor, which would provide a metabolic advantage under conditions such as high availability of ethanolamine.

### 4.4. Limitations of the Study

Of the 25 samples processed for the 16S rRNA gene profiling analysis, four did not yield sequence data, and were excluded from downstream analyses. While the authors acknowledge that this missing data could have introduced a bias in the clustering results, it also reinforces the importance of the metagenomic analyses in providing complementary data that supported the results from the 16S rRNA gene profiling. Additionally, the authors recognize that further investigations are needed to validate adenosylcobalamin (Ado-Cbl) biosynthesis and ethanolamine utilization in the candidate bacterial species identified in this study, which would require to isolate the organisms using culturing techniques, then quantify their response to elevated lipid concentrations.

## 5. Conclusions

This report highlighted the metabolic potential of rumen bacteria that were found to be prominent under conditions of high dietary triglyceride content. Certain features of these uncharacterized species were expected, such as lipase or esterase activity, the potential for glycerol utilization, as well as biohydrogenation; other shared metabolic activities, however, were not as easily predicted, such as ethanolamine utilization and the biosynthesis of vitamin B_12_. In the case of this study, dietary triglycerides may have favored ruminal vitamin B_12_ synthesizers, which in turn would have impacted the rumen microbiota composition by promoting the growth of vitamin B_12_ salvagers. Further research on vitamin B_12_ cross-feeding in the rumen could provide valuable insights into optimizing lipid inclusion in ruminant diets, as well as serve as a model system for investigating the impact of microbial specialists on gut microbial ecology.

## Figures and Tables

**Table 1:** Metabolic pathways of interest and the number of enzymes identified in the respective contig sets assigned to each OTU.

	Enzymes Detected (per OTU/Total)				
Metabolic Pathways	Bt-995 ^1^	Bt-1367 ^1^	Bt-1391 ^1^	Key Product(s) ^2^	Reference ^3^
Glycolysis	7/10 (70.0%)	9/10 (90.0%)	6/10 (60.0%)	Pyruvate	[43]
Non-oxidative Pentose Phosphate Pathway	5/8 (62.5%)	7/8 (87.5%)	2/10 (20.0%)	PRPP	[44]
Purine metabolism	19/38 (50.0%)	20/38 (52.6%)	16/38 (42.1%)	GTP	Supplementary Materials S2
Histidine metabolism	4/9 (44.4%)	7/9 (77.8%)	0/9 (0.0%)	Histidine	[45,46]
De novo Riboflavin synthesis	2/9 (22.2%)	8/9 (88.9%)	0/9 (0.0%)	Riboflavin (B_2_), FMN, FAD	Supplementary Materials S2
De novo Adenosylcobalamin synthesis	15/28 (53.6%)	20/28 (71.4%)	0/28 (0.0%)	Adenosyl-cobalamin, Methyl-cobalamin	Supplementary Materials S2

^1^ The numerator indicates the number of enzymes detected in the contig set, while the denominator represents the total number of enzymes expected for that pathway. Percent completeness is also provided in parentheses. ^2^ The “Key Products” column lists the major metabolites or building blocks derived from each pathway. ^3^ References correspond to the sources used to define pathway enzyme totals.

**Table 2:** Enzyme counts for de novo vitamin B_12_ biosynthesis stages in the respective contig set of each OTU.

Stages of the Vitamin B_12_ Biosynthesis Pathway	Bt-1367 ^1^	Bt-995 ^1^	Bt-1391 ^1^	EC Numbers
1. Tetrapyrrole precursor biosynthesis (5 total)	4/5.	0/5	0/5	ALA synthesis (either EC:2.3.1.37 or both EC:1.2.1.70 and EC:5.4.3.8), EC:4.2.1.24, EC:2.5.1.61, EC:4.2.1.75, EC:2.1.1.10722
2. Combined corrin ring biosynthesis (10-anaerobic pathway total)	8/10	8/10	0/10	EC:2.1.1.151, EC:2.1.1.131, EC:2.1.1.271, EC:3.7.1.12 and EC:2.1.1.195, EC:1.3.1.106, EC:2.1.1.289, EC:2.1.1.196, EC:5.4.99.60, EC:6.3.5.11, and EC:4.99.1.3.
3. Aminopropanol Linker	1/2	1/2	0/2	EC:2.7.1.177, EC:4.1.1.81
4. Adenosylation	1/1	0/1	0/1	EC:2.5.1.17
5. Nucleotide loop assembly (7 total)	6/7	7/7	0/7	EC:6.3.5.10, EC:6.3.1.10, EC:2.7.1.156, cobinamide activation (EC:2.7.7.62), cobalamin phosphatase (EC:3.1.3.73), EC:2.7.8.26
Key Enzymes from Shelton et al. [70]	^2^			
Core biosynthesis genes (8 total)	8/8.	8/8.	0/8	(EC:2.1.1.130 or EC:2.1.1.151), (EC:2.1.1.133 or EC:2.1.1.271), (EC:5.4.99.61 or EC:5.4.99.60), EC:6.3.5.10, EC:6.3.1.10, EC:2.7.1.156, cobinamide activation (EC:2.7.7.62), EC:2.7.8.26

^1^ Values represent detected enzymes/total expected enzymes for each pathway stage. Core biosynthesis genes denote the essential set required for complete de novo vitamin B_12_ synthesis. ^2^ The lower section highlights the key enzymes described by Shelton et al. [70] found in >99% of B_12_-capable organisms [70].

**Table 3:** Classification of OTUs based on the criteria described by Shelton et al. (2019) [70] for vitamin B_12_ biosynthesis potential.

OTU	Bt-1367	Bt-995	Bt-1391
Classification ^1^	possible cobamide producer	cobinamide (cbi) salvage	very likely non-producer
Explanation according to Shelton 2019 [70]	≥6/9 corrin ring biosynthesis steps and (either ≥18/25 anaerobic steps or ≥16/23 aerobic steps) or ≥16/21 (tetrapyrrole precursor + corrin ring + nucleotide loop assembly steps)	not any of the producer categories nor tetrapyrrole precursor salvager and has ≥5 nucleotide loop assembly steps	not any of the complete biosynthesis categories or partial biosynthesis categories and has ≤5/9 corrin ring steps

^1^ OTUs were categorized as possible cobamide producers, cobinamide (cbi) salvagers, or likely non-producers, according to the presence of corrin ring biosynthesis genes and the completeness of associated pathway steps. Detailed classification thresholds are provided in Shelton et al. (2019) [70].

## Data Availability

The raw sequence data presented in the study are openly available in NCBI Sequence Read Archive at https://dataview.ncbi.nlm.nih.gov/object/PRJNA1322392?reviewer=tpuasknmtv8v6b8sg64rn6t4qh (accessed on 9 November 2025) under BioProject PRJNA1322392.

## Supplementary Materials

The following supporting information can be downloaded at https://www.mdpi.com/article/10.3390/microorganisms13112617/s1, Supplementary Material S1: Latin square design and sample-treatment key for the original animal study; Supplementary Material S2: In-house references of the metabolic pathways based on KEGG; Supplementary Material S3: Relative proportion of the 20 most abundant OTU by sample; Supplementary Material S4: Cluster dendrogram of ruminal samples based on 16S rRNA gene profiling; Supplementary Material S5: Manually curated mapping of CAZyme families to substrates for representative OTUs; Supplementary Material S6: List of Vitamin B12 ATP-binding cassette (ABC) transporters associated with the contig set of each OTU; Supplementary Material S7: Known vitamin B_12_-dependent enzymes and their B_12_-independent homologs; Supplementary Table S1: Metabolic pathways of interest and the number of enzymes identified in each OTU; Supplementary Table S2: Enzyme counts for de novo vitamin B_12_ biosynthesis stages in each OTU; Supplementary Table S3: Classification of OTUs based on vitamin B_12_ biosynthesis potential; Supplementary Figure S1: Heatmap showing the relative abundance of the top 20 OTUs across samples; Supplementary Figure S2: Ethanolamine utilization complex; Supplementary Figure S3: Anaerobic de novo biosynthesis of vitamin B_12_ (adenosylcobalamin, Ado-Cbl).
